# Autophagy in *Tenebrio molitor* Immunity: Conserved Antimicrobial Functions in Insect Defenses

**DOI:** 10.3389/fimmu.2021.667664

**Published:** 2021-05-31

**Authors:** Yong Hun Jo, Jung Hee Lee, Bharat Bhusan Patnaik, Maryam Keshavarz, Yong Seok Lee, Yeon Soo Han

**Affiliations:** ^1^ Department of Applied Biology, Institute of Environmentally-Friendly Agriculture (IEFA), College of Agriculture and Life Sciences, Chonnam National University, Gwangju, South Korea; ^2^ P. G. Department of Biosciences and Biotechnology, Fakir Mohan University, Balasore, India; ^3^ Department of Evolutionary Biology, Institute for Biology–Zoology, Free University of Berlin, Berlin, Germany; ^4^ Department of Biology, College of Natural Sciences, Soonchunhyang University, Asan City, South Korea

**Keywords:** *Tenebrio molitor*, Listeria monocytogenes, autophagy, NF-kappaB, innate immunity

## Abstract

The yellow mealworm beetle (*Tenebrio molitor*) has been exploited as an experimental model to unravel the intricacies of cellular and humoral immunity against pathogenic infections. Studies on this insect model have provided valuable insights into the phenotypic plasticity of immune defenses against parasites and pathogens. It has thus been possible to characterize the hemocoelic defenses of *T. molitor* that rely on the recognition of non-self-components of pathogens by pattern recognition receptors (PRRs). The subsequent signaling cascade activating pathways such as the NF-κB controlled by Toll and IMD pathways lead to the synthesis of antimicrobial peptides (AMPs), onset of hemocyte-driven phagocytosis, and activation of the prophenoloxidase cascade regulating the process of melanization. Nevertheless, the activation of autophagy-mediated defenses of *T. molitor* against the facultative intracellular gram-positive bacterium *Listeria monocytogenes* provides clear evidence of the existence of a cross-talk between autophagy and the IMD pathway. Moreover, the identification of several autophagy-related genes (*Atgs*) in *T. molitor* transcriptome and expressed sequence tag (EST) databases has contributed to the understanding of the autophagy-signaling cascade triggered by *L. monocytogenes* challenge. Providing further evidence of the cross-talk hypothesis, *TmRelish* has been shown to be required not only for regulating the synthesis of AMPs through the PGRP-LE/IMD pathway activation but also for the expression of *Atgs* in *T. molitor* larvae following *L. monocytogenes* challenge. Notably, *L. monocytogenes* can stimulate the *T. molitor* innate immune system by producing molecules recognized by the multifunctional PRR (*Tm*PGRP-LE), which stimulates intracellular activation of the IMD pathway and autophagy. Considering the conservation of autophagy components involved in combating intracellular pathogens, it will be interesting to extrapolate a dynamic cross-talk model of immune activation. This review summarizes the most significant findings on the regulation of autophagy in *T. molitor* during *L. monocytogenes* infection and on the role of the innate immunity machinery, including the NF-κB pathway, in the control of pathogenic load.

## Introduction

Autophagy is well-known as a conserved cellular mechanism by which the cell degrades unnecessary and/or dysfunctional cellular components through the action of lysosomes. It maintains homeostasis during cellular stress and pathogen or infective organism invasion. For instance, under nutrient starvation, oxidative stress, and intracellular pathogen invasion, the autophagic machinery is activated in order to detoxify cells and maintain a surveillance of cellular components for intensified cell function (1, 2). Further, in coordination with apoptosis regulator Bcl-2, the autophagy protein Beclin-2 inhibits apoptosis in normal physiological and pathological conditions (3).

**Figure 1:**
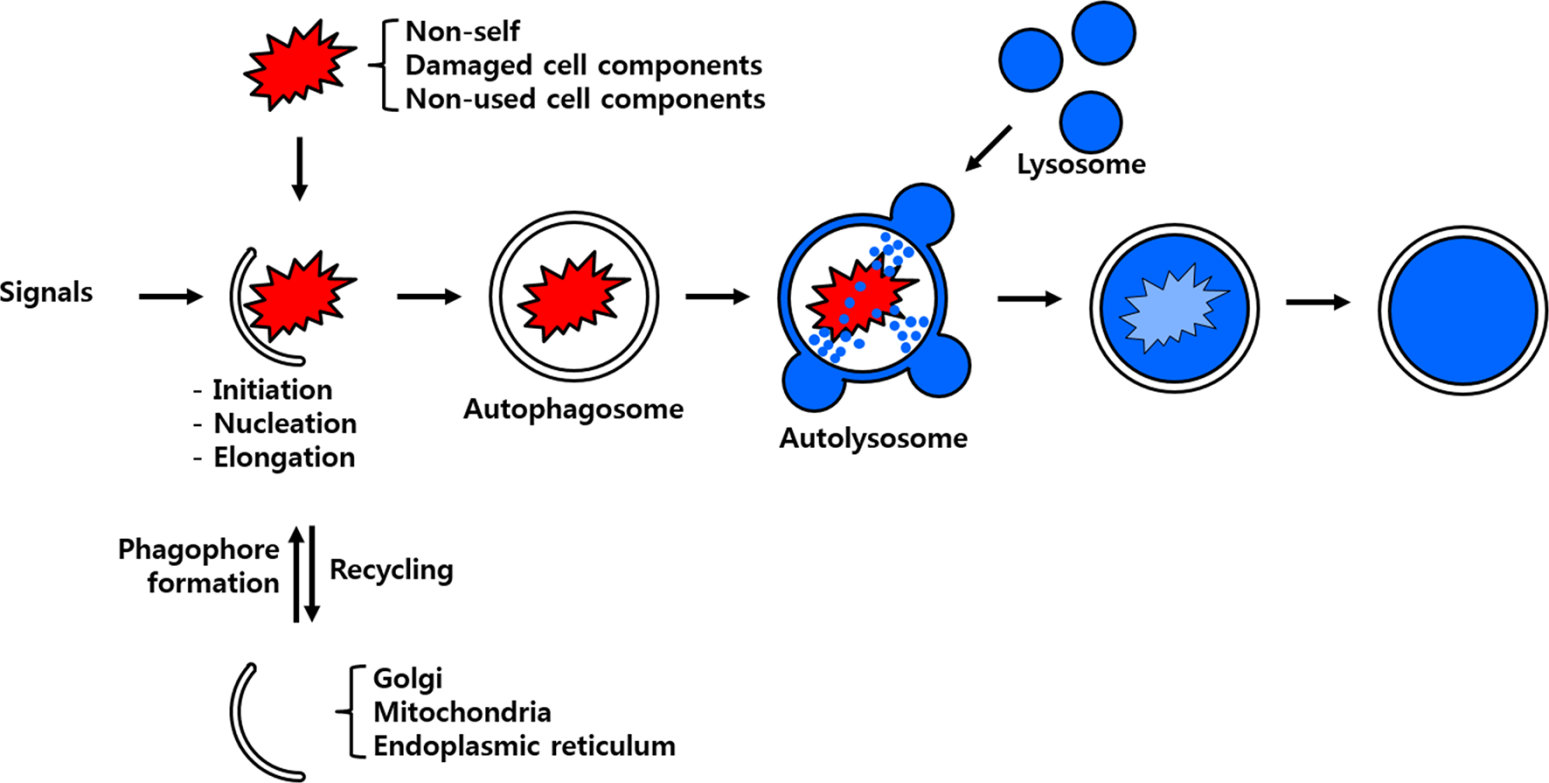
Scheme of the process of autophagy. Non-self-entities such as pathogenic bacteria and unused or damaged cell components are enclosed by membrane particles believed to stem from the Golgi apparatus, mitochondria and/or endoplasmic reticulum. The developing autophagosome fuses with the lysosome to deliver its cargo to hydrolytic enzymes for eventual degradation and/or recycling.

There are three known forms of autophagy, namely microautophagy, macroautophagy and chaperone-mediated autophagy (CMA). Although all the autophagy forms culminate in the lysosomal degradation of cellular cargo including viruses intracellularly they are distinguished from one another on the basis of the pathway by which the cargo is delivered to the lumen of the autolysosome (4). Microautophagy is initiated by a direct and random invagination of a membrane around a portion of the cytoplasm that subsequently differentiates into an autophagic tube to enclose portions of the cytosol (5). The much well-studied and conserved macroautophagy mechanism in eukaryotes requires the formation of a double-membrane vesicular structure called the autophagosome (6). In this process, targeted cellular components are isolated from the rest of the cellular cytosolic components within the newly developing autophagosome. Subsequently, the autophagosome fuses with the lysosome to form the autolysosome in which the enclosed cellular components are degraded and/or recycled (**Figure 1**). In yeast, the autophagosome formation is mediated by the hierarchical recruitment of autophagy-related (Atg) proteins to the phagophore assembly site or preautophagosomal structure (PAS). In contrast, PAS-like structure have not been identified in mammals, wherein multiple cellular organelles serve as origins for the assembly of the phagophore (7, 8). Consequent upon the recruitment of Atg proteins, the phagophore gets extended to form an autophagosome. Upon maturity, the autophagosome are transported along the endocytic pathway before fusing with the lysosomes to form autophagolysosome. Subsequently, the cellular cargos are degraded by the hydrolytic enzymes of lysosomes and degradation products are released back to the cytoplasm for cell use (9). Viruses such as the Severe acute respiratory syndrome coronavirus 2 (SARS-CoV-2) escape the macroautophagy regulatory cascade as the viral protein ORF3a block the fusion of the autophagosome to the lysosome, thus evading eventual degradation (10). With the exception of SARS-CoV-2, the autophagy regulatory cascade involving *Atg* genes have been found to be receptive to vesicular stomatitis virus (VSV), Rift Valley fever virus (RVFV) or Zika virus infection in *Drosophila* model (11, 12). In contrast, the dengue virus (DENV-2) titers in mosquito *Aedes aegypti* Aag2 cells were not affected in *Atg* silenced individuals suggesting diverse autophagic response in insects (13). In CMA, all proteins containing the penta-peptide motif ‘KFERQ’ in their amino acid sequences are selectively recognized by a specialized cytosolic chaperone known as the heat shock cognate protein of 70 kDa (HSC70) (14). The resulting complex is targeted to the lysosomal membrane where it binds to a receptor called lysosome-associated membrane protein type 2A (LAMP-2A) (5). The presence of a luminal form of HSC70 (lys-HSC70) is required for the complete translocation of the chaperone-associated target protein complex into the lysosomal lumen for its eventual degradation.

In this review, we focused on the regulatory autophagy signaling cascade (illustrating macroautophagy) in the yellow mealworm, *Tenebrio molitor* and its putative role in the host during *Listeria monocytogenes* infection. Further, a cross-talk mechanism of autophagy process and the NF-κB pathway in *T. molitor* in response to invasion of infective microorganisms have been suggested.

## Mechanism and Regulation of Autophagy

Macroautophagy (here-after referred to as autophagy) is a highly regulated process that can be conveniently divided into three major steps: autophagy induction (or initiation), vesicle nucleation, and vesicle expansion (or elongation) and completion.

### Autophagy Induction and Formation of the Initiation Complex

**Figure 2:**
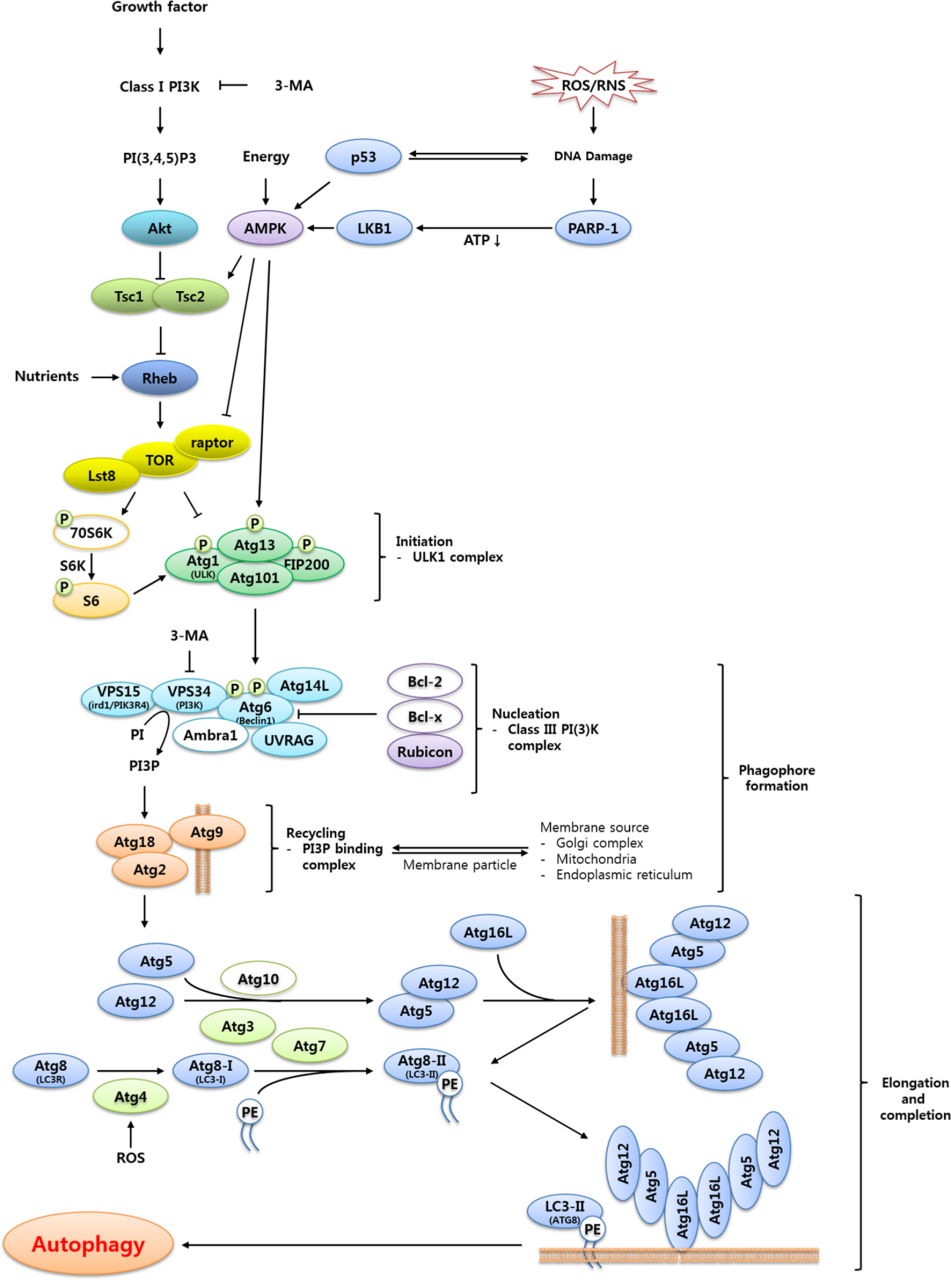
Proposed signaling cascade triggering TOR-regulated autophagy in *T. molitor.* Most of the autophagy-related genes included in the scheme were identified in the *T. molitor* RNA-Seq and EST databases. Growth factors such as the insulin receptor and nutrient-rich conditions negatively regulate autophagy, while reactive oxygen/nitrogen species (ROS/RNS), DNA damage and a low cellular energy status (that is, a high AMP/ATP ratio) induce autophagy. Upon generation of autophagy signals by the appropriate stimuli, an initiation complex comprising Atg1, Atg13, Atg101 and Fip200 is formed. Subsequently, the nucleation complex consists of the Vps15-Vps34-Atg6-Atg14-UVRAG complex, alternatively named Class III PI (3)K complex. In addition, the Atg2-Atg18 complex supports the delivery of the membrane particles from the Golgi, mitochondria or endoplasmic reticulum. Vesicle elongation is carried out by two ubiquitin-like complexes: the Atg5-Atg12:Atg16 complex, generated through the E1- and E2-like enzymatic activity of Atg7 and Atg10, respectively; and the Atg8-PE complex, generated by the proteolytic activity of Atg4 before transfer of Atg8 to the E1- and E2-like enzymes Atg7 and Atg3, respectively. Open circles show either non-Atg components (3-MA, PI3P, Class I PI3K, PI(3,4,5)P3 and PE) or autophagy related genes (70S6K, Bcl-2, Bcl-x, Ambra1 and Atg10) unidentified in *T. molitor*.

The induction of autophagy is triggered by a variety of stress cues that include, but are not limited to, nutrient deprivation, hypoxia, endoplasmic reticulum (ER) stress, and oxidative stress (15). In most cases, these stimuli trigger autophagy induction through activation of the nutrient energy sensor AMP-activated protein kinase (AMPK) or inhibition of the negative regulator of autophagy target of rapamycin (TOR) (15). Among the several pathways mediating the transmission of autophagic signals, the TOR-dependent autophagic pathway is relatively well-characterized. The detailed description of TOR-dependent autophagic pathway is discussed in **Figure 2**.

In eukaryotes, autophagy induction is achieved through Atg1 [insect homolog of unc-51 like autophagy activating kinase 1 (ULK1)] complex formation, following inactivation of its upstream negative regulator TOR (**Figure 2**). However, there are significant differences in the composition and function of the core components of the Atg1 complex among yeast, mammals, and insects (16). In yeast, for example, Atg1 interacts with at least eight other Atg proteins and TOR regulates the formation of Atg1-Atg13-Atg17 complex (17). Conversely, humans and *Drosophila* do not possess clear orthologs of Atg17 or its interacting proteins Atg29 and Atg31 (16, 18, 19). Instead, the mammalian Atg1 ortholog ULK1 forms a complex with Atg13 and two additional proteins Atg101 and the focal adhesion kinase family-interacting protein of 200 kDa (FIP200), both of which are required for autophagosome formation. Furthermore, while TOR-mediated phosphorylation of Atg13 must be suppressed to allow Atg13 to interact with and activate Atg1 in yeast, the mammalian and *Drosophila* orthologs of Atg13 are associated with ULK1. Moreover, in *Drosophila*, Atg13 becomes hyper-phosphorylated upon autophagy induction (20). In addition, overexpression of *Drosophila* Atg1 has been shown to induce autophagy; whereas overexpression of ULK1 inhibits autophagy (21, 22). The proposed explanations for this difference include the influence of additional regulatory proteins and the existence of feedback regulation between Atg1 and TOR (16, 17).

### Vesicle Nucleation

Vesicle nucleation starts with the recruitment of yeast Atg proteins, or their equivalents in higher animals, to the PAS. The mechanism regulating this process is not yet clear, but the activation of the class III phosphatidylinositol 3-kinase (PI3K) complex is indispensable (20). In fact, following autophagy induction by the activated Atg1/ULK1 complex, a structure enriched in phosphatidylinositol-3-phosphate [PI3P, also known as PtdIns (3)P] appears at the site of autophagosome formation (16).

PI3P is synthesized by enzymes of the PI3K family, which phosphorylate the 39-hydroxyl group on the inositol ring of phosphoinositides (23). Three classes of PI3K enzymes are known: class I enzymes are composed of p100 catalytic subunits and p85 adaptors: class II enzymes are large (> 200 kDa) and characterized by a C2 domain at the C-terminus; finally, class III enzymes are homologous to the vacuolar protein sorting 34 (Vps34), the only PI3K characterized in yeast (23). In yeast, Vps34 can form complexes with other autophagic components such as Atg6, Atg14, and Vps15. The mammalian orthologs of such proteins have been named Vps34, Beclin-1, Atg14, and p150, respectively (24).

In higher animals, complexes formed by class I PI3Ks are considered negative regulators of autophagy. For example, the activation of class I PI3Ks by the insulin receptor leads to phosphorylation of plasma membrane lipids, which in turn recruit and activate Akt/protein kinase B (PKB), a downstream negative regulator of autophagy (25). In contrast, complexes formed by class III PI3Ks are divided into several types depending on the proteins that interact with the core components, namely Atg6 or Beclin, Vps34 and Vps15 (20). Proteins such as UV-resistance associated gene (UVRAG), activating molecule in Beclin-1-regulated autophagy (AMBRA1), Atg14L, and Bax-interacting factor-1 (Bif-1) are known to positively regulate autophagy when interacting with the core complex proteins. Conversely, other proteins interacting with class III PI3Ks such as run domain Beclin-1- interacting and cysteine-rich containing protein (Rubicon), Cln-2, and Bcl-x_L_ are negative regulators of autophagy (16, 24, 26, 27).

Although PI3P production is essential for autophagosome formation, its exact role in phagophore formation is yet to be deciphered. It has been proposed that PI3P production is involved in altering the composition of the ER-derived lipid bilayer to create a phagophore or simply in the recruitment of PI3P-binding proteins that are required for the synthesis of the phagophore (28). For instance, the autophagy proteins Atg18 and Atg21 are known to be recruited by PI3P to the developing phagophore. Moreover, in yeast, Atg18 can form a complex with Atg2, which localizes to the PAS depending on the PI3P-binding ability of Atg18 (29).

The appearance of a developing phagophore is crucial for the completion of the vesicle nucleation step. However, the exact source of the developing phagophore is unknown. Nevertheless, two proposed models have received considerable scientific support. The first model suggests that the phagophore is synthesized by a *de novo* mechanism and expands through the addition of lipids from other sources, transported to the site by Atg9 (30). The second model suggests that the initial cradle for the formation of the phagophore is derived from a subdomain of the ER and later expands between the ER domains to which it is physically linked (31, 32). However, there is an increasing body of literature that implicates several other intracellular organelles, including the Golgi apparatus, endosomes, mitochondria and the plasma membrane as sources of initial building material for the phagophore (16, 33–35). The mechanism for determining the site of phagophore formation within the cytosol of higher eukaryotes is also not well understood. In yeast, the PAS functions as an organizing center for autophagosome formation because most of the Atg proteins necessary for the formation of the developing phagophore are usually recruited to the PAS (16). It is not clear, however, if an equivalent of the PAS does exists in higher eukaryotes.

### Vesicle Expansion and Completion

In the canonical autophagy machinery, vesicle expansion and completion involve at least eight Atg proteins. These proteins are grouped into two major sets according to their functional participation in ubiquitin-like conjugation reactions. In particular, Atg5-Atg12/Atg16 complex requires Atg7 and Atg10 as E1- and E2-like enzymes, respectively. Similarly, Atg8-[known as microtubule-associated protein 1 light chain 3 (LC3) in mammals]-phosphatidylethanolamine (PE) complex requires the proteolytic activity of Atg4 together with the E1- and E2-like enzymatic activity of Atg7 and Atg3, respectively. The *Drosophila* genome contains two orthologs of the yeast *Atg*8, denoted as *Atg8a* and *Atg8b*. Similarly, the human genome includes four orthologs with high sequence identity to the yeast *Atg4*.

The regulation of vesicle expansion and completion is mediated by the activity of two highly conserved ubiquitin-like protein complexes, namely Atg5-Atg12/Atg16 complex and Atg8-PE complex. In the Atg5-Atg12/Atg16 complex, Atg12 is a ubiquitin-like protein containing a C-terminal glycine residue. Atg12 is first activated by the E1-like enzyme Atg7, which transfers it to the E2-like enzyme Atg10. In turn, Atg10 catalyzes the covalent binding of the C-terminal glycine residue of Atg12 to a lysine residue of Atg5 (36). The Atg12-Atg5 complex then associates non-covalently with Atg16; such interaction is required for the localization of the Atg12-Atg5 complex to the PAS (37, 38).

The formation of the Atg8-PE complex is accomplished through the conjugation of the ubiquitin-like protein Atg8 to the head-group of a membrane PE lipid molecule. Atg8 is normally synthesized with a C-terminal arginine residue masking the penultimate C-terminal glycine residue. Due to the proteolytic activity of Atg4, such arginine is removed, thus exposing the glycine residue to the activity of the ubiquitin-like enzyme. The cleaved Atg8 is then transferred to Atg7, which in turn transfers Atg8 to Atg3, an E2-like enzyme. Next, Atg3 transfers Atg8 to a membrane PE. The resulting Atg8-PE complex is thought to mediate membrane tethering, thereby assisting the expansion of the isolation membrane through the promotion of vesicle carrier fusion (39). In particular, the inhibition of the Atg8 conjugation system in mammalian cells has been shown to lead to failure of isolation -membrane closure into a complete autophagosome (38), suggesting that Atg8 is responsible for the late step of autophagosome formation.

**Figure 3:**
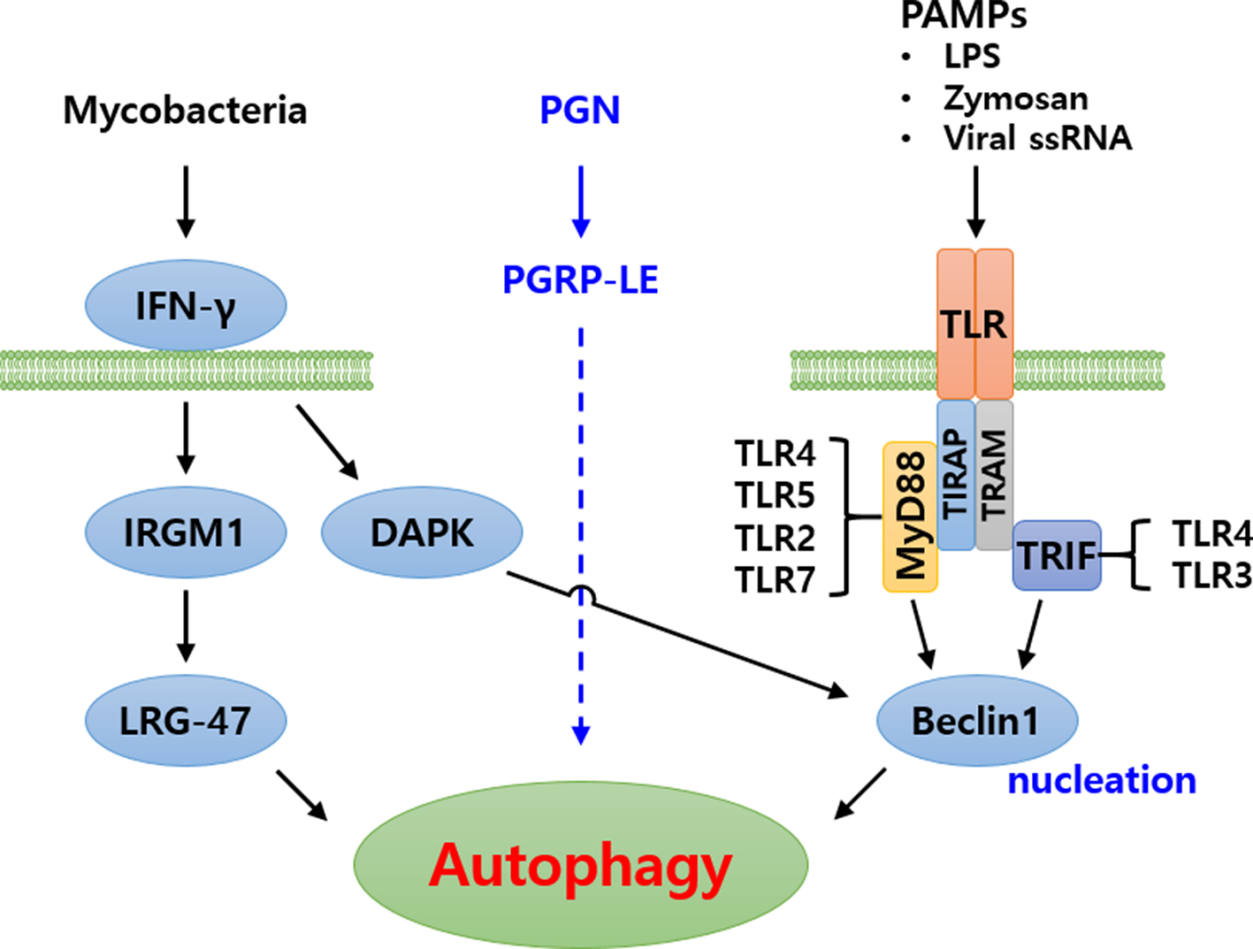
Schematic representation of autophagy signaling cascade induced by pathogenic bacteria in humans. Three major signaling cascades have been identified. In the first, Mycobacteria are recognized by IFN-γ, subsequently inducing autophagy. In the second, bacterial peptidoglycans (PGN) are recognized by PGRP-LE and induce autophagy. The signaling cascade is yet to be identified. In the third cascade, Pathogen associated molecular patterns (PAMPs) are recognized by Toll-like receptor forming Toll-MyD88-TIRAP complex or Toll-TRIF-TRAM complex, intracellularly. These complexes subsequently signal to Beclin1 (Atg6) for induction of autophagy.

Based on the above-mentioned findings, it is noted that the hierarchical functioning of several Atg proteins are crucial towards regulation of autophagic machinery in eukaryotes. In addition to the extensively studied yeast autophagy model, considerable work on autophagy has been carried out in mammalian and *Drosophila* model systems (16). The autophagy pathway induced by various pathogens (Mycobacteria and virus) and PAMPs (PGN, LPS and ssRNA) has been nicely illustrated in humans (**Figure 3**) (40, 41). However, autophagy research in the beetle, *T. molitor* is still in its infancy. Therefore, an attempt has been made to review the identification and characterization of Atg protein orthologs in *T. molitor* and the role during host-pathogen interactions.

## Conservation of the Autophagy System in *T. molitor*

The sequences of the *Atg* genes of *Drosophila* were used as query against unpublished RNA sequencing (RNA-Seq) and expressed sequence tag (EST) sequence databases of *T. molitor* to identify *Atg* genes in *T. molitor* (42). After confirmation of search results *via* local-TBLASTN analysis, the candidate *T. molitor Atg* genes were grouped based on their functional location along the autophagy signaling pathway. The genes were thus placed within the categories “upstream signaling”, “initiation of autophagy”, “vesicle nucleation”, and “vesicle expansion and completion”.

### Upstream Genes Regulating Autophagy Induction in *T. molitor*

A total of 13 genes putatively involved in the regulation of autophagy induction upstream of/or in conjunction with TOR, a central cell growth regulator that integrates signals from growth factors and nutrient dynamics (43), were identified in *T. molitor*. These candidate genes are presented along with their orthologs in humans, yeast (*Saccharomyces cerevisiae* S288c) and *Drosophila* in **Table 1**. Except for liver kinase B1 (*Lkb1*) and *p53*, which were only detected in the *T. molitor* EST database, the rest other genes were identified in both the *T. molitor* RNA-Seq and EST databases. Notably, TOR is present in two copies (*TOR1* and *TOR2*) in the genome of yeast, whereas only one copy of this gene has been characterized in humans*, Drosophila*, and *T. molitor*. Furthermore, putative orthologs of all other genes of *Drosophila* and humans belonging to the category, “upstream regulatory genes” were identified in *T. molitor*. However, homologs of genes such as *p53*, poly-(ADP-ribose) polymerase (*Parp*), Tuberose sclerosis 1 (*Tsc1*) and *Tsc2* have not been characterized in yeast, despite having been identified from humans and *T. molitor*.

**Table 1:** Up-stream autophagy signaling genes in *T. molitor*.

Generic name	*Homo sapiens*			*Saccharomyces cerevisiae* S288c			*Drosophila melanogaster*			*Tenebrio molitor*		
	Specific name	Accession No.	Amino acid length	Specific name	Accession No.	Amino acid length	Specific name	Accession No.	AminoAcid length	Specificname	Amino acid Lengths (Tm/Tc)^*^	Source
*Akt1*	*Akt1*	AAL55732.1	480	–	–	–	*Akt1*	NP_732114.1	530	*Akt1*	515(F)^**^/ 510	EST, RNA-Seq
AMP-activated protein kinase (*AMPK*)	*AMPK*	AAB32732.1	552	AMPK gamma	P12904.1	322	*SNF1A*	NP_477313.1	582	*AMPK*	526(F)^**^/526	EST, RNA-Seq
liver kinase B1 (*LKB1*)	*LKB1*	AAB05809.1	433	–	–	–	*LKB1*	NP_650302.1	567	*LKB1*	458(F)^**^/443	EST
Lethal with Sec Thirteen 8 (*Lst8*)	*Lst8*	AAO73410.1	260	Lst8	NP_014392.3	303	*Lst8*	NP_572572.1	313	*Lst8*	309(F)^**^/309	RNA-Seq
*P53*	*p53*	BAC16799.1	393	–	–	–	*p53*	NP_001247252.1	318	*p53*	375(F)^**^/350	EST
Poly-(ADP-ribose) polymerase (*Parp*)	*Parp1*	NP_001609.2	1,014	–	–	–	*Parp*	NP_001104452.1	994	*Parp*	983(P)^**^/991	RNA-Seq
Regulatory-associated protein of mTOR (Raptor)	*Raptor*	AAM09075.1	1,335	Mitochondrial DNA polymerase-encoding 1 (Mip1p)	NP_014975.2	1,254	*Raptor*	NP_572294.2	1,621	*Raptor*	1,273(F)^**^/1,264	RNA-Seq
Ras homolog enriched in brain (*Rheb*)	*Rheb*	NP_005605.1	184	Rheb1p	NP_009956.2	209	*Rheb*	NP_730950.2	182	*Rheb*	182(F)^**^/182	EST, RNA-Seq
Ribosomal Protein S6 (*RpS6*)	*S6*	NP_001001.2	249	–	–	–	*RpS6*	NP_511073.1	248	*RpS6*	248(F)^**^/248	EST, RNA-Seq
Ribosomal protein S6 kinase (*S6k*)	*S6K*	NP_003152.1	525	ribosomal 40S subunit protein S6B	DAA07295.1	236	*S6k* (CG10539)	NP_523941.2	490	*S6K*	746(F)^**^/738	RNA-Seq
Target of rapamycin (TOR)	*mTOR*	NP_004949.1	2,549	Tor1p	NP_012600.1	2,470	*TOR*	NP_001260427.1	2,471	*TOR*	2,397(F)^**^/2,400	RNA-Seq
				Tor2p	NP_012719.2	2,474						
Tuberous sclerosis 1 (*TSC1*)	hamartin	NP_001155898.1	1,163	–	–	–	*Tsc1*	NP_477415.1	1,100	*TSC1*	1,056(F)^**^/1,047	RNA-Seq
Tuberous sclerosis 2 (*TSC2*)	*TSC2*	AAI50301.1	1,784	–	–	–	gigas	NP_524177.1	1,847	*TSC2*	1,735(F)^**^/1,722	RNA-Seq

*Amino acid lengths have been predicted based on the length of T. molitor (Tm) and T. castaneum genes (Tc) genes.

**(F) and (P) indicate full-length open reading frame (ORF) cDNA sequences, respectively.

Genes putatively involved in the generation and processing of the signals that eventually activate the master regulator TOR. Most of them were identified in the T. molitor RNA-Seq database (but only a few in the EST database) using the Drosophila orthologs as a reference. A comparison of the identified genes with their homologs in the unicellular eukaryote S. cerevisiae and the multicellular human (H. sapiens) is given. Blank boxes indicate that no homologs of the respective genes, especially those of S. cerevisiae, were found.

### Genes Involved in Autophagy Initiation in *T. molitor*

The composition of the autophagy initiation complex varies among eukaryotes although two of its components, the yeast Atg1 and Atg13, have orthologs conserved in both insects and mammals. A total of four genes putatively required for the formation of the initiation complex in higher eukaryotes, namely *Atg1*, *Atg13*, *Atg101* and *FIP200*, were identified by screening the *T. molitor* RNA-Seq database (**Table 2**). Orthologs of all these candidate genes were also found in representative higher eukaryotes such as *Drosophila* and humans.

**Table 2:** Autophagy-related genes involved in autophagy initiation in *T. molitor*.

Generic name	*Homo sapiens*			*Saccharomyces cerevisiae* S288c			*Drosophila melanogaster*			*Tenebrio molitor*			
	Specific name	Accession No.	Amino Acid length	Specific name	Accession No.	Amino Acid length	Specific name	Accession No.	Amino Acid length	Specific name	Amino Acid Lengths (Tm/Tc)^*^	Source
Autophagy-specific gene 1 (*Atg1*)	unc-51 like autophagy activating kinase 1 (*ULK1*)	NP_003556.1	1,050	*Atg1p*	NP_011335.1	897	*Atg1*	NP_001163433.1	855	*Atg1*	792(F)^* *^/779	RNA-Seq
Autophagy-specific gene 13 (*Atg13*)	*Atg13*	NP_001192048.1	550	*Atg13p*	NP_015511.1	738	*Atg13*	NP_649796.1	523	*Atg13*	391(F)^**^/399	RNA-Seq
Autophagy-specific gene 29 (*Atg29*)	–	–	–	*Atg29p*	NP_015159.1	213	*-*	–	–	*-*	–	–
Autophagy-specific gene 31 (*Atg31*)	–	–	–	*Atg31p*	NP_010305.1	196	–	–	–	*-*	–	–
Autophagy-specific gene 101 (*Atg101*)	*Atg101*	NP_068753.2	218	*-*	–	–	CG7053	NP_573326.1	218	*Atg101*	220(F)^**^/220	RNA-Seq
FAK family-interacting protein of 200 kDa (*Fip200*)	RB1-inducible coiled-coil 1 (*RB1CC1*)	NP_055596.3	1,594	*Atg17p*	NP_013527.3	417	CG1347	NP_649573.2	1357	*Fip200*	1,387(F)^**^/1,382	RNA-Seq

*Amino acid lengths have been predicted based on the length of T. molitor (Tm) and T. castaneum genes (Tc) genes.

**(F) and (P) indicate full-length ORF cDNA sequences and partial ORF cDNA sequences, respectively.

Genes involved in the formation of the autophagy initiation complex. Following activation of TOR in response to up-stream autophagic signals, a signaling cascade is activated by the relief of TOR-dependent suppression, culminating in the formation of the initiation complex. The genes involved in the assembly of the initiation complex are Atg1 and Atg13, together with Atg17, Atg29, and Atg31 in yeast or Atg101 and Fip200 in higher eukaryotes, including T. molitor.

Two additional genes required for the formation of the initiation complex in yeast*, Atg29 and Atg31* (16) were not characterized in higher eukaryotes including *T. molitor*. Similarly, *Atg17*, whose protein product is required for the formation of the initiation complex in yeast, is not known to have clear orthologs in higher eukaryotes. However, a non-orthologous functionally equivalent gene, *FIP200*, existing in both *Drosophila* and humans was also identified in *T. molitor*.

### Genes Involved in Vesicle Nucleation in *T. molitor*

In yeast, vesicle nucleation during autophagy is achieved through the regulatory activity of a complex consisting of Vps34 and three other proteins: Vps15, Vps30/Atg6 and Atg14. The mammalian orthologs of these proteins have been identified and named Vps34, p150, Beclin-1 and Atg14 (24). Orthologs of these proteins have also been identified in the RNA-Seq database of *T. molitor*. Putative orthologs of UVRAG and Rubicon, which is part of a Beclin-1-Vps34-containing autophagy complex in mammals (44), have also been identified in *T. molitor*. Furthermore, orthologs of the PI3P–binding protein Atg18 and its interaction partner Atg2, required for autophagosome formation in yeast, *Drosophila* and humans, were also identified in *T. molitor* (**Table 3**). Additionally, we also identified *T. molitor* Atg9, a multi-spanning membrane protein supporting the supply of lipid bilayers required for the formation of the autophagosome in yeast and mammalian systems (45, 46). Notably, the *AMBRA1, Bcl-2* and *Bcl-x* regulatory genes were not identified in the *T. molitor* RNA-Seq database. These three genes have also not been identified in the genome of yeast (**Table 3**).

**Table 3:** Autophagy-related genes involved in vesicle nucleation in *T. molitor*.

Generic name	*Homo sapiens*			*Saccharomyces cerevisiae* S288c			*Drosophila melanogaster*			*Tenebrio molitor*		
	Specific name	Accession No.	Amino acid length	Specific name	Accession No.	Amino acid length	Specific name	Accession No.	Amino acid length	Specific name	Amino acid lengths (Tm/Tc)^*^	Source
Activating molecule in Beclin1-regulated autophagy (*AMBRA1*)	*AMBRA1*	ABI74670.1	1,269	–	–	–	–	–	–	–	–	–
Autophagy-specific gene 2 (*Atg2*)	*Atg2a*	NP_055919.2	1,938	*Atg2p*	NP_014157.1	1,592	*Atg2*	NP_647748.1	1906	*Atg2*	1,945(P)^***^/2,040	RNA-Seq
	*Atg2b*	NP_060506.5	2,078									
Autophagy-specific gene 6 (*Atg6*)	Bcl-2 interacting coiled-coil protein (*Beclin1*)	AAD27650.1	450	*Vps30p*	NP_015205.1	557	*Atg6*	NP_651209.1	422	*Atg6*	386(F)^***^/396	RNA-Seq
Autophagy-specific gene 9 (*Atg9*)	*Atg9a*	NP_001070666.1	839	*Atg9p*	NP_010132.1	997	*Atg9*	NP_001261023.1	852	*Atg9*	718(F)^***^/662	RNA-Seq
	*Atg9b*	NP_775952.4	924									
Autophagy-specific gene 14 (*Atg14*)	*Atg14*	NP_055739.2	492	*Atg14p*	NP_009686.1	344	*CG11877*	NP_651669.1	503	*Atg14*	472(F)^***^/478(Bt)^**^	RNA-Seq
Autophagy-specific gene 18 (*Atg18*)	WD repeat domain, Phosphoinositide interacting 1 (*WIPI1*)	NP_060453.3	446	*Atg18p*	NP_444297.1	500	*Atg18*	NP_648184.1	377	*Atg18*	406(F)^***^/409	RNA-Seq
B-cell leukemia/ lymphoma-2-alpha protein (*Bcl-2*)	*Bcl-2*	ABX60202.1	239	–	–	–	*Debcl*	NP_788278.1	300	–	–	–
*Bcl-x*	*Bcl-x*	AAB17354.1	227	–	–	–	–	–	–	–	–	–
Run domain Beclin-1- interacting and cystein-rich containing protein (Rubicon)	*KIAA0226*	NP_001139114.1	927	–	–	–	–	–	–	*Rubicon*	880(F)^***^/884	EST
UV-resistance associated gene (*Uvrag*)	*UVRAG*	BAA90829.1	699	–	–	–	*Uvrag*	NP_609632.1	696	*UVRAG*	729(F)^***^/716	RNA-Seq
Phosphoinositide 3-kinase regulatory subunit 4 (*PIK3R4*)	*PIK3R4*	NP_055417.1	1,358	Vacuolar protein sorting 15 (*VPS15p*)	NP_009655.2	1,454	Immune response Deficient 1 (*ird1*)	NP_649868.1	1342	*VPS15*	1,337(F)^***^/1,330	RNA-Seq
Phosphotidyl-inositol 3 kinase (*PI3K*)	*PIK3C3*	NP_002638.2	887	Vacuolar protein sorting 34 (*VPS34p*)	NP_013341.1	875	Phosphatidyl-inositol 3 kinase 59F (*Pi3K59F*)	NP_477133.1	949	*VPS34*	1,700(P)^***^/2,070	RNA-Seq

*Amino acid lengths have been predicted based on the length of T. molitor (Tm) and T. castaneum genes (Tc) genes.

**Bt; Amino acid lengths based on Bombus terrestris gene.

***(F) and (P) indicate full-length ORF cDNA sequences and partial ORF cDNA sequences

This step involves the recruitment of Atg proteins to the phagophore assembly site (PAS) in yeast or its equivalents in higher animals. Various Atg proteins taking part in this process were identified in the RNA-Seq database.

### Genes Involved in Vesicle Expansion and Completion in *T. molitor*

As detailed earlier, two ubiquitin-like protein complexes (the Atg5-Atg12/Atg16 and Atg8-PE complexes) and four important enzymes, namely the proteolytic enzyme Atg4, the E1-like enzyme Atg7, and the E2-like enzymes Atg3 and Atg10, are required for vesicle expansion and completion. The eight corresponding genes were highly conserved in all experimental species, from yeast to humans. Of note, four isotypes of *Atg4* genes and two isotypes of the *Atg8* have been identified in humans and *Drosophila*, respectively. Putative orthologs of the autophagy genes involved in vesicle expansion and completion were identified in the *T. molitor* RNA-Seq and EST databases, except for the gene encoding Atg10, acting as an E2-like enzyme for the assembly of the Atg5-Atg12/Atg16 complex (**Table 4**).

**Table 4:** Autophagy related genes involved in elongation and completion in *T. molitor*.

Generic name	*Homo sapiens*			*Saccharomyces cerevisiae* S288c			*Drosophila melanogaster*			*Tenebrio molitor*		
	Specific Name	Accession No.	Amino acid length	Specific name	Accession No.	Amino acid length	Specific name	Accession No.	Amino acid length	Specific name	Amino acid lengths (Tm/Tc)^*^	Source
Autophagy-specific gene 3 (*Atg3*)	*Atg3*	NP_071933.2	314	*Atg3p*	NP_014404.3	310	*Aut1*	NP_649059.1	330	*Atg3*	320(F)^***^/316	EST, RNA-Seq
Autophagy-specific gene 4 (*Atg4*)	*Atg4a*	AAH61696.1	398	*Atg4p*	NP_014176.2	494	*Atg4*	NP_608563.1	411	*Atg4*	369(F)^***^/366	RNA-Seq
	*Atg4b*	EAW71278.1	415									
	*Atg4c*	EAX06579.1	458									
	*Atg4d*	AAH68992.1	474									
Autophagy-specific gene 5 (*Atg5*)	*Atg5*	AGC52703.1	275	*Atg5p*	NP_015176.1	294	*Atg5*	NP_572390.1	269	*Atg5*	263(F)^***^/263	RNA-Seq
Autophagy-specific gene 7 (*Atg7*)	*Atg7*	NP_006386.1	703	*Atg7p*	NP_012041.1	630	*Atg7*	NP_611350.1	684	*Atg7*	623(F)^***^/621	RNA-Seq
Autophagy-specific gene 8 (*Atg8*)	microtubule-associated protein 1 light chain 3 alpha (*MAP1LC3A*)	NP_852610.1	125	*Atg8p*	NP_009475.1	117	*Atg8a*	NP_727447.1	121	*Atg8*	120(F)^***^/120	RNA-Seq
							*Atg8b*	NP_650649.1	120			
Autophagy-specific gene 10 (*Atg10*)	*ATG10*	NP_001124500.1	220	*Atg10p*	NP_013058.1	167	*CG12821*	NP_001097216.1	212	*-*	–	–
Autophagy-specific gene 12 (*Atg12*)	*Atg12*	ACD74941.1	187	*Atg12p*	NP_009776.1	186	*Atg12*	NP_648551.3	111	*Atg12*	129(F)^***^/124	RNA-Seq
Autophagy-specific gene 16 (*Atg16*)	*Atg16*	NP_110430.5	607	*Atg16p*	NP_013882.1	150	*CG31033*	NP_733313.2	604	*Atg16*	544(F)^***^/552(Bt)^**^	EST, RNA-Seq

*Amino acid lengths have been predicted based on the length of T. molitor (Tm) and T. castaneum (Tc) genes.

**Bt; amino acid lengths predicted based on the lengths of Bombus terrestris (Bt) gene.

***(F) and (P) indicate full-length ORF cDNA sequences and partial ORF cDNA sequences, respectively.

## Autophagic Signatures of *T. molitor* in Response to the Intracellular Bacterium, *Listeria Monocytogenes*

*T. molitor* has been exploited as an experimental model for host-pathogen interaction studies, especially those to unravel the intricacies of the insect innate immunity. In the last decade, owing to the availability of whole genome, transcriptome, and EST sequences, researchers in the field were able to explore the intricacies of cellular and humoral immunity against pathogenic infections in this model insect. In particular, elegant biochemical, molecular, and *in silico* studies allowed to identify the components of the *T. molitor* Toll and IMD signaling cascades and to propose their role in insect immunity (47, 48). The conserved role of both extracellular and intracellular pathwaypathway components have been elucidated through protein induction, gene expression and RNA interference (RNAi)-based gene silencing studies (49–51).

Most significantly, valuable insights into humoral immunity were obtained through the study of the synthesis of antimicrobial peptides (AMPs), hemocyte-driven phagocytosis, and the prophenoloxidase cascade regulating the process of melanization (52). In the context of autophagic control of *T. molitor* defenses, the RNA-Seq and EST databases have contributed to unravel the mysteries of the autophagy-signaling cascade upon *L. monocytogenes* challenge. Further, in some recent studies, the existence of a cross-talk between autophagy and the NF-κB-controlled IMD pathway upon *L. monocytogenes* challenge in *T. molitor* has been advocated (53); this could help extrapolate general mechanism describing the regulation of host immune strategies against pathogenic microorganisms. In the next paragraphs, we review the components of the conserved autophagy signaling cascade in *T. molitor* and show that they are functionally required to counteract the pathogenicity of *L. monocytogenes*. We not only lay emphasis on the role of *Atgs* in beetle immunity, with special reference to autophagy-based clearance of *Listeria* in the *T. molitor* model, but also delineate reasonable hypotheses on the extent of the cross-talk between autophagy and the IMD pathway.

In separate studies, we have functionally characterized TOR (54), the HORMA-domain-containing protein Atg13 (55), Atg6 (Beclin-1 in humans) (56), Atg3/Atg5 (42), and Atg8 [microtubule-associated protein 1 light chain 3 alpha (MAPILC3A) in humans] (57) in the *T. molitor* model. The corresponding genes have been listed together with other *T. molitor Atgs* in **Table 5** and have been categorized, due to their conserved functions, as upstream autophagy signaling genes (*TmTOR*), autophagy-related genes involved in autophagy initiation (*TmAtg13*), autophagy-related genes for vesicle nucleation (*TmAtg6*), and autophagy-related genes for elongation and completion of autophagosome formation (*TmAtg3, TmAtg5*, and *TmAtg8*). Specifically, the autophagy machinery of *T. molitor* has been functionally dissected in the process of *L. monocytogenes* clearance or suppression by the host. Notably, *L. monocytogenes*, a facultative gram-positive bacterium, has been used for the study of host-pathogen interactions in both vertebrates and invertebrates (58).

**Table 5:** Summary of the autophagic signatures functionally characterized in *T. molitor* implicated in autophagy-mediated clearance of the gram-positive bacterium *L. monocytogenes*.

Autophagy genes	Autophagy functional category	Nucleotide and protein features			Role in autophagy in relation to *L. monocytogenes* infection	Reference
		Nucleotide length (ORF)	Protein length	Domain analysis		
*Atg3*	Elongation and completion of autophagosome formation	963 bp	320 aa	N-terminal domain, catalytic (autophagy-related protein 3) domain and C-terminal domain	Putative role in mediating autophagy-based clearance of *Listeria* in the *T. molitor* model	(42)
*Atg5*	Elongation and completion of autophagosome formation	792 bp	263 aa	Autophagy-related protein 5 domain	Putative role in mediating autophagy-based clearance of *Listeria* in the *T. molitor* model	(42)
*Atg13*	Initiation (ULK1 complex)	1,176 bp	391 aa	N-terminal Atg13 domain with a HORMA (Hop1, Rev7 and Mad2 fold)	Not studied	(55)
*Atg8* (Atg8-II/PE complex)	Elongation and completion of autophagosome formation	363 bp	120 aa	Microtubule-associated proteins 1a/1b light chain B-related domain	Putative role in autophagy-based clearance of *Listeria* in *T. molitor*	(57)
Target of rapamycin (*TOR*)	Upstream signaling genes	7,197 bp	2,398 aa	Huntington domain, EF3A, ATM, TOR (HEAT) repeat, and focal adhesion kinase targeting (FAT), rapamycin binding, phosphatidylinositol 3-/4-kinase, and FRAP, ATM and TRRAP C-terminal (FATC) domains	Negative correlation with autolysosome formation after bacterial challenge	(54)
*Atg6*	Nucleation (class III PI(3)K complex)	1,161 bp	386 aa	Atg6 domain	Putative role in autophagy-based clearance of *Listeria* in *T. molitor*	(56)

*A cross-talk mechanism between the IMD and autophagy pathways is established as TmRelish, a transcription factor of the IMD pathway is implicated not only in the regulation of AMP genes but also in the induction of autophagy genes in response to L. monocytogenes challenge. In fact, TmAtg1 was downregulated both in hemocytes and fat body upon TmRelish knockdown

In *Drosophila*, the autophagy machinery is stimulated after recognition of *L. monocytogenes* invasion by PGRP-LE, whereas transcription factors downstream of the NF-κB pathway (Relish, Dif, and Dorsal) and Atg5 are not required for PGRP-LE-dependent suppression of *L. monocytogenes* growth (59). Similar to the *Drosophila* model, the requirement of *Tm*PGRP-LE for defense of *T. molitor* against *L. monocytogenes* infection was established using a gene silencing assay (60). In fact, knockdown of *TmPGRP-LE* followed by *L. monocytogenes* challenge led to a marked reduction in the survival of *T. molitor* larvae, thereby clearly indicating that *Tm*PGRP-LE is a fundamental component of the pathogen recognition system, which could modulate the downstream NF-κB signaling cascade or the autophagic machinery to eliminate or suppress the pathogenicity of microorganisms.

**Figure 4:**
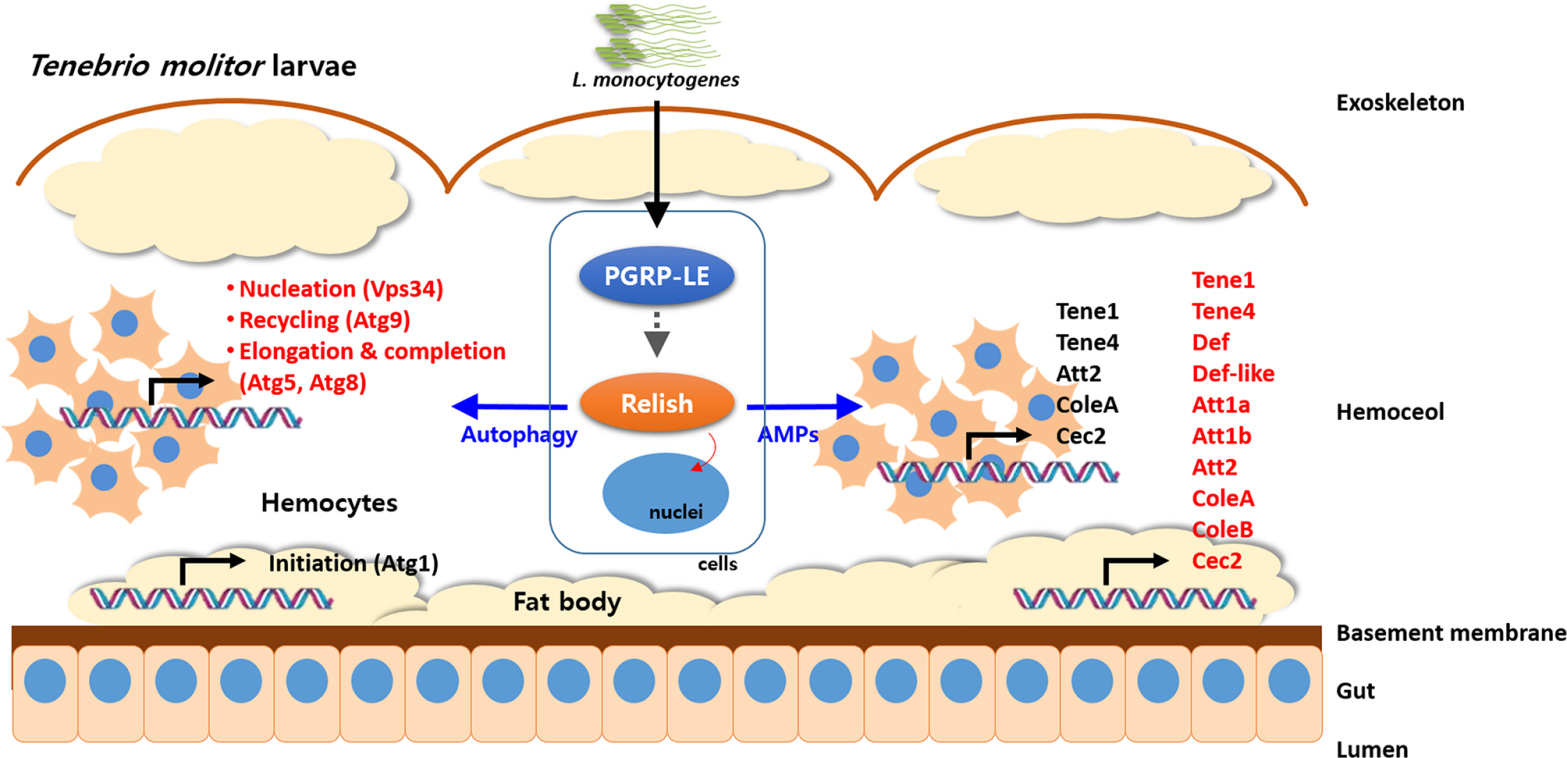
Schematic illustration depicting cross-talk of autophagy process and NF-κB (Relish) pathway in response to pathogens in *T. molitor*. The intracellular Gram-positive bacterium, *Listeria monocytogenes* is recognized by PGRP-LE and the signaling cascade activates the NF-κB transcription factor, Relish. Subsequently, Relish regulates activation of autophagy by direct or indirect stimulation of nucleation step in hemocytes and AMP production in fat body. In addition, the Atg genes related to the nucleation (*TmVPS34*), recycling (*TmAtg9*), and elongation and recycling (*TmAtg5* and *TmAtg8*) were significantly regulated by silencing of *TmRelish* in hemocytes.

Although it is logical to assume that the *L. monocytogenes-*induced autophagy is independent of the IMD pathway in *Drosophila*, the existence of a cross-talk between autophagy and the IMD pathway seems more of a reasonable hypothesis. In this regard, we have observed that knocking down *TmRelish*, encoding a transcription factor downstream of the PGRP-LE/IMD pathway, could directly or indirectly influence the expression of AMP and *Atg* genes after *L. monocytogenes* challenge in the fat body and hemocytes of *T. molitor* larvae (**Figure 4**). Ultimately, *L. monocytogenes* infection led to reduced survival of *TmRelish*-knockdown *T. molitor* larvae. While assessing the expression of *Atg* genes, it was found that the mRNA levels of *TmAtg1* were significantly decreased in the fat body and hemocytes of *T. molitor* larvae after *L. monocytogenes* infection (53). Atg1/ULK-1 is related to autophagy initiation, and its increased expression level is necessary to initiate an appropriate autophagic response in *Drosophila* and *Bombyx mori* (22, 61). Furthermore, the formation of the Atg1/Atg13 complex leads to the nucleation of the autophagosome membrane, thereby possibly promoting autophagy. Although *Drosophila* Atg13 has been reported to enhance both the pro-autophagic activity of Atg1 and the inhibition of TOR signaling, the role of Atg13 in the molecular mechanisms underlying autophagy initiation in other insects has been studied to a lesser extent. For instance in *Bombyx mori*, Atg13 (*BmAtg13*) knockdown and overexpression have been implicated in autophagy inhibition (62). In fact, overexpression of *BmAtg13* gene promotes the replication and proliferation of *B. mori* nucleopolyhedrovirus (*Bm*NPV) and silencing results in suppression of *Bm*NPV replication (63). *TmAtg13* is implicated in the survival of the host to both *Escherichia coli* and *Staphylococcus aureus* infection, since *TmAtg13*-silenced larvae exhibited reduced survival under microorganism challenge (55).

Atg8/MAP1LC3A is a ubiquitin-like protein controlling the expansion of the phagophore during autophagosome formation. Moreover, it is a reliable marker of autophagosome formation, and its expression during autophagy has been studied in various insect species. In *Galleria mellonella*, increased activity of Atg8 in parallel to autophagosome formation has been associated with the perivisceral fat body remodeling (64). The Atg8/LC3 positive hemocytes in *G. mellonella* 24 and 48 hours post-infection with entomopathogenic fungus, *Conidiobolus coronatus* also relate to autophagosome formation and autophagy (65). Further, in the hematophagous insect *Rhodnius prolixus*, silencing of *Atg8* transcripts led to disrupted lipophagy (sequestration of lipid droplets and degradation of triacylglycerol generating free fatty-acids for β-oxidation) (66). In *Drosophila*, elevation of Atg8-II level and accumulation of Atg8 in the autophagic punctae have been demonstrated after infection with VSV, RVFV or Zika virus as evidenced through silencing experiments (67, 68). In *Laodelphax striatellus*, Atg8 facilitates Rice stripe virus (RSV) infection in an autophagy-independent manner (69). The higher mortality rates in *Aedes albopictus* mosquitoes after blood-feeding of microorganisms and not systemic challenge in *Atg8*-silenced hosts suggest modulation of autophagy in gut immunity (70). The role of the Atg8 homolog of *T. molitor* (*Tm*Atg8) in mediating autophagy-based clearance of *L. monocytogenes* has been demonstrated through dsRNA-induced gene silencing and recombinant protein expression analysis. Furthermore, the expression levels of *TmAtg8* in hemocytes were also found to be markedly reduced after *L. monocytogenes* infection. In contrast, the increasing expression level of autophagy genes with time in *L. monocytogenes*-infected larvae injected with *dsEGFP* (negative control for RNAi) suggested autophagic control of *L. monocytogenes* clearance (57). Another member of the Atg8 family, the microtubule-associated proteins 1A/1B light chain 3C-like protein (*Tm*Lc3), exhibiting low amino acid identity (34%) to *Tm*Atg8 has been characterized in *T. molitor*; however, its exact role in autophagy-based immunity is yet to be deciphered. Moreover, *Tm*Atg8 protein expression failed to be induced in hemocytes of *TmAtg5*- knockdown larvae, suggesting a coordinated functional role of elongation and completion of autophagosome assembly for the clearance of *L. monocytogenes* in the host. Consistently, RNAi-mediated loss of function of *TmAtg5* led to impaired formation of the Atg12-Atg5-Atg16 complex, which facilitates the lipidation of Atg8, that is, the conversion of Atg8-I to Atg8-II, to form the Atg8-PE complex. Further, the lack of induction of the autophagy marker *Tm*Atg8-II in the fat body tissues of *T. molitor* following *L. monocytogenes* infection is interesting and warrants detailed studies for understanding the mechanism of preferential establishment of autophagy in certain tissues following *L. monocytogenes* challenge. Certainly, the increase in Atg8 (Atg8-II) signals overtime after exposure to *L. monocytogenes* infection is indicative of an increasing autophagic control in the host. The involvement of Atg8 in *Leishmania* parasite survival while infecting macrophages *in vitro* suggests regulatory control of autophagy by autophagosome formation (71).

In another study, the requirement of *Tm*Atg3 and *Tm*Atg5 for the autophagic control of *Listeria* infection was thoroughly investigated. RNAi-mediated silencing of *TmAtg3* and *TmAtg5* led to an increased susceptibility of *T. molitor* larvae to *L. monocytogenes* infection, further confirming the requirement of Atg3 and Atg5 for Atg8 lipidation, formation of the Atg8-PE complex, and subsequent activation of autophagic clearance of microorganisms (42). While *Tm*Atg3 is an E2 ubiquitin-like enzyme responsible for covalent binding of PE to the C-terminus of Atg8, Atg5 is part of the Atg12-Atg5-Atg16 complex, which regulates the formation of the Atg8-PE complex. Downregulation of *Atg8* transcript also downregulating *Atg3* expression in insects is suggestive of an interconnection between the two genes (72). *Tm*Atg6 (Beclin-1 in mammals), involved in the vesicle nucleation step of the autophagy pathway, has also been found to be required to protect *T. molitor* larvae against *L. monocytogenes* challenge (56). Although Atg6 is a multifunctional protein involved in sorting of vacuolar contents, pollen germination, and tumor suppression, its role in autophagy is highly conserved. In the autophagic pathway, Atg6 forms a complex with Vps34, Vps15, UVRAG, and Vps38 to generate the phagophore. Hence, Atg6 supports autophagy-mediated immune responses against *L. monocytogenes*.

## Summary Statement

Genes involved in the canonical TOR-regulated autophagy pathway, which were reviewed here, can be conveniently divided into categories depending on their presumed location along the pathway. Upstream regulatory genes in the *T. molitor* autophagy pathway include the master- regulator *TOR* and at least seven genes, namely, *Akt1*, *Lkb1*, *p53*, *Parp*, *RpS6*, *Tsc1*, and *Tsc2*, that appear to be absent in the yeast genome are conserved in higher eukaryotes such as *Drosophila* and humans. Most of these genes have been reported to be active regulators of autophagy induction and tumor suppressors in model organisms. Similarly, the gene sets required for the formation of the autophagy initiation complex (Atg1 kinase complex) after successful induction vary between yeast and higher eukaryotes. For instance, the absence of Atg17 and its interacting proteins Atg29 and Atg31 in higher eukaryotes including *T. molitor* highlights the discrepancy in the identity of the core components of the autophagy initiation complex. Indeed, in higher eukaryotes Atg17 seems to have been mechanistically replaced by a non-homologous functional equivalent, Fip200 (73). Adding to this complexity, the protein Atg101, absent in yeast interacts with both Atg1 and Fip200 to form the Atg1-kinase complex in higher eukaryotes.

The variation in the composition and function of the core autophagy machinery between yeast and higher eukaryotes discussed above highlights the fact that, although genetic screening in *S. cerevisiae* laid the foundation for the molecular understanding of autophagy, fundamental differences in active components and processes exist between yeast and higher eukaryotes. Indeed, the autophagy core machinery of higher eukaryotes has been extensively modified, partly to satisfy the fundamental need to account for the greater genetic diversity and complexity of higher eukaryotes with respect to those of unicellular eukaryotes. Such difference is substantiated by the presence of multiple isoforms of certain *Atg* genes, including *Atg4*, *Atg8*, *Atg2*, and *Atg9* in higher eukaryotes, although no evidence of this rare phenomenon in *S. cerevisiae* was found in *T. molitor* (74).

## Future Perspectives

The early discovery of Atg8 lipidation in the 1990s prompted researchers to focus on the mechanisms and functional roles of autophagy-related proteins; in addition, recent studies have raised considerable interest in a wide range of research directions to dissect the autophagy pathway. Current findings on the autophagy pathway in insect model systems derived from various fields such as developmental biology, immunology, epidemiology, and molecular biology, have improved our understanding of the fundamental framework of the autophagy process. In particular, the *T. molitor Atg* genes have been largely identified, and the functional roles of these signaling components have been demonstrated through gene silencing studies. However, many unanswered questions on the importance of autophagy in metamorphosis and starvation of *T. molitor*, as well as the role of the interaction between Atg proteins and membrane lipids remains to be answered. Importantly, recent studies in *T. molitor* and *Drosophila* have revealed the regulation of *Atg* genes through NF-κB factors, suggesting a possible connection between different pathways of immune response, including the IMD pathway and autophagy. In *Drosophila*, while the activation of autophagy by the plasma membrane receptor Toll-7 is independent of NF-κB response during VSV infection, the autophagic response to Zika virus infection is NF-κB-dependent (67, 68, 75). Despite the significance of altered *NF-κB* expression at the transcriptional level, the levels of the corresponding protein level seems to be critical in *T. molitor*. Further, it would be interesting to note the levels of conservation in the transcription factors inhibiting autophagy induction such as Hox-family of proteins in *Drosophila* and the zinc finger with a SCAN and KRAB domain 3 (ZKSCAN3) in humans. With transcriptome signatures of pathogenic responses in the host readily available in the research domain, a high-throughput unraveling of Atg genes and its regulatory roles in autophagy would become a reality. In fact, the transcriptome of *An. aquasalis* midgut epithelium is implicated to trigger an autophagic response to *Plasmodium vivax* invasion (76). In conclusion, the fascinating results obtained from previous studies on *T. molitor* autophagy has established promising future directions for newcomers and experts in this field of research.

## Author Contributions

YJ and YH: Design manuscript concepts. YJ and JL: wrote the draft manuscript. YJ, BP, MK, and YH: Wrote the manuscript. YH and YL: revised the manuscript. All authors contributed to the article and approved the submitted version.

## Funding

This research was supported by Korea Institute of Planning and Evaluation for Technology in Food, Agriculture, Forestry and Fisheries (IPET) through Export Promotion Technology Development Program (Grant no. 617077‐5), funded by Ministry of Agriculture, Food, and Rural Affairs (MAFRA).

## Conflict of Interest

The authors declare that the research was conducted in the absence of any commercial or financial relationships that could be construed as a potential conflict of interest.
